# Evaluating and Modeling the Internal Diffusion Behaviors of Microencapsulated Rejuvenator in Aged Bitumen by FTIR-ATR Tests

**DOI:** 10.3390/ma9110932

**Published:** 2016-11-17

**Authors:** Junfeng Su, Yingyuan Wang, Peng Yang, Shan Han, Ningxu Han, Wei Li

**Affiliations:** 1 Tianjin Key Laboratory of Advanced Fibers and Energy Storage, Tianjin Polytechnic University, Tianjin 300387, China; weili007@163.com; 2 Department of Polymer Materials, School of Material Science and Engineering, Tianjin Polytechnic University, Tianjin 300387, China; yywang@163.com (Y.W.); shanhan@163.com (S.H.); 3 School of Navigational Engineering, Guangzhou Maritime Institute, Guangzhou 510725, China; pyang@163.com; 4 Guangdong Provincial Key Laboratory of Durability for Marine Civil Engineering, Shenzhen University, Shenzhen 518060, China

**Keywords:** diffusion behaviors, bitumen, self-healing, microcapsule, rejuvenator

## Abstract

Microencapsulated rejuvenator has been attracted much attention for self-healing bitumen. The diffusion coefficient is one of the key parameters to estimate the feasibility of rejuvenator to age bitumen. The objective of this research was to evaluate diffusion behaviors of microencapsulated rejuvenator in aged bitumen by a FTIR-ATR method. Various microcapsule samples were mixed in bitumen to form thin films. The core material of microcapsules used as rejuvenator was diphenylsilane (DPS), its fairly specific absorption band at 843 cm^−1^ was selected as a marker band to calculate the diffusion coefficient (D). The microstructure parameters, including contents, mean size and mean shell thickness of microcapsules, were considered to understand the diffusion behaviors under different temperatures (20, 30, 40 and 50 °C) in bitumen. The results showed that a larger mean size of microcapsules did not greatly affect the D values under the same temperature. In contrast, a higher mean shell thickness decreased the D values because of the decrement of damage probability of microcapsules under the same content. With the same microcapsule sample in bitumen, the D values presented a trend of linear increase when the content of microcapsules was increased. All these results indicated that the microstructure affected the diffusion behaviors based on the concentration of released rejuvenator. A preliminary model of diffusion behaviors of microencapsulated rejuvenator in bitumen was given based on the Arrhenius equation considering the microstructure of microcapsules, the amount of released rejuvenator and the age degree of bitumen. This model may be a guide to the construction and application of self-healing bitumen using microcapsules.

## 1. Introduction

Bitumen can be defined as a self-healing material because it has the potential to restore stiffness and strength by closing the microcracks which occur when the pavement is subjected to traffic loads or under high temperature [1]. Its self-healing capability will decrease or disappear as the ageing problem of bitumen leads to pavement failure after years of usage including surface raveling and reflective cracking [2]. Rejuvenator use may be the only one method that can restore the original properties of the pavements because rejuvenating agents have the capability of reconstituting the binder’s chemical composition and consist of lubricating and extender oils containing a high proportion of maltene constituents [3]. To overcome the poor penetration ability of oily rejuvenator through the surface of bitumen, microencapsulated rejuvenator in situ use in bitumen may be an alternative approach [4]. In previous study, a method has been reported to fabricate microcapsules containing rejuvenator utilizing methanol modified melamine-formaldehyde (MMF) resin as shell material [5,6,7,8,9,10]. This product is believed to be an environmental-friendly powder encapsulating suitable size rejuvenator for chemical engineering and construction engineering [4]. During the aging process of bitumen, microcapsules could be punctured by microcracks and leaked the oily-liquid rejuvenator into microcracks [8]. The mechanism is that a crack repair in an asphalt pavement system attributes to the wetting and inter-diffusion of material between the two surfaces of a microcrack to achieve properties of the original material [10]. With the help of capillarity, the rejuvenator filled the cracks with a movement speed mainly determined by the volume of microcapsules in bitumen [8]. A diffusion phenomenon was also observed by using a fluorescence microscope [8,9,10]. During the diffusion behaviors, the small molecules will insert into the macromolecules of bitumen, and then the microstructure will be changed. It was found that the diffusion behaviors made a role of determining the microstructure of bitumen [9].

Rejuvenating products are designed to restore the original characteristics to oxidised (aged) bitumen binders in order to soften the aged binder and create a broad-spectrum rejuvenation that replenishes the volatiles and dispersing oils while promoting adhesion. Therefore, diffusion is an important concept considering processes in bituminous binders such as oxidative ageing, stripping and rejuvenator in asphalt recycling [11]. A literature review shows that diffusion in bitumen has attracted much research. A number of issues related to diffusion in bitumen have been investigated by Karlsson et al. [12]. For example, consequences of Fick’s law governing the diffusion process were demonstrated, which, among other things, indicated that the time needed for a diffusion process to occur was proportional to the square of the binder layer thickness [13]. Factors influencing diffusion, such as the temperature, ageing and refining of bitumen (bitumen obtained from one and the same crude oil from various degrees of distillation) have also been studied [13]. Normally, the diffusing capability of rejuvenator into aged bitumen can be enhanced with the increasing of temperature and time, however, the diffusing of rejuvenator into aged bitumen is restricted due to the volatilization of light component and aging of rejuvenator under high temperature [11,12,13].

At the same time, a few methods have been reported to measure the diffusion rules in bitumen. For example, atomic force microscope (AFM) and gel permeation chromatography (GPC) were used to analyze the diffusion mechanism of different kinds of rejuvenator [14]. The result showed the rejuvenator influence gradually weakened when the diffusion depth grew. Adsorbed rejuvenator in age asphalt hindered the diffusion of large molecular modifying component by dispersing the asphaltenes. The diffusion level of the modifying rejuvenator closed to that of low viscosity regeneration. These research results provide technical support to the regeneration road performance. In our previous work [9,10], capillarity and diffusion behaviors of rejuvenator in aged bitumen were observed by a fluorescence microscope using light characters such as reflection, diffraction and refraction. The scale of fluorescence microscope was applied to measure the size of capillarity and diffusion. As bitumen is a temperature-sensitive material, the observation is in an environment of 0 °C temperature. The defect of this testing method is its low precision limited by observer vision. Fourier transform infrared spectroscopy with attenuated total reflectance (FTIR-ATR) was used to monitor the diffusion of selected well-defined substances [12]. The penetration depth of rejuvenator seal materials on hot asphalt mixtures was studied by means of rheology test and FTIR test. Karlsson [13] proved that the FTIR-ATR was a reliable method to measure the rejuvenator diffusion in bitumen. The advantages of FTIR-ATA method include the in situ measurement, non-destructive measurement and real-time measurement.

**Figure 1:**
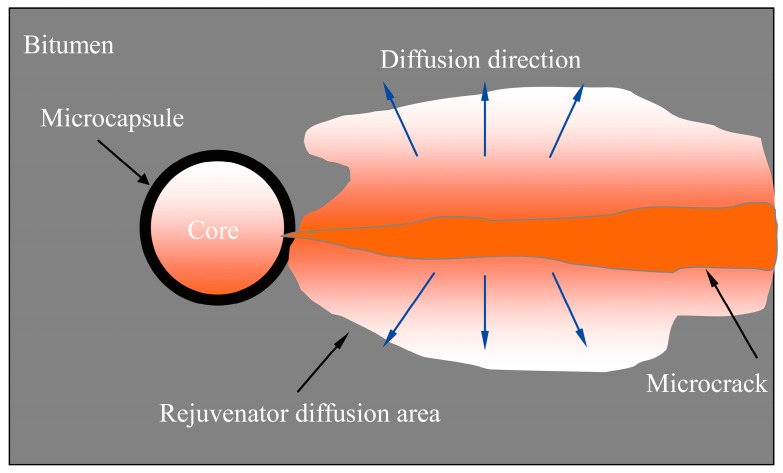
Illustration of the diffusion process of microencapsulated rejuvenator into bitumen.

Although several methods have been used to measure diffusion behaviors of rejuvenator into bitumen, little knowledge can be applied to monitor the internal diffusion principle of microencapsulated rejuvenator in bitumen. Figure 1 illustrates the diffusion process of microencapsulated rejuvenator into bitumen. The diffusion dynamics is the concentration difference of rejuvenator material in bitumen. One reason is that it is difficult to measure the internal diffusion occurring in microcracks with a micrometer size. Another reason is that it is hard to measure the diffusion behaviors because of less mass of rejuvenator in microcapsules. Furthermore, the microstructure of microcapsules (shell thickness and mean size) will greatly influence the rejuvenator movements [6]. It has been hypothesized that the temperature and microstructure of bitumen may be critical for the diffusion process, as diffusion is a molecule’s movement, but it is not yet clear how it may affect it.

Based on the above analysis, the objective of this research is to evaluate diffusion behaviors of microencapsulated rejuvenator in aged bitumen by a FTIR-ATR method. Various contents of microcapsules were mixed in bitumen to form thin film samples. Liquid nitrogen was used to trigger a microcrack in bitumen samples. Fluorescence microscope was used to observe the diffusion behaviors. FTIR-ATR was applied to measure the diffusion rates affected by time, temperature, microcapsules content, and age degree of bitumen. A preliminary diffusion model was given for the microcapsules/bitumen samples considering the parameters of microstructure, time and temperature. The diffusion principles are expected to guide the optimization of this intelligent material systems and structures.

## 2. Experimental Method

### 2.1. Materials

The shell material of microcapsules was commercial prepolymer of melamine-formaldehyde modified by methanol (solid content was 78.0%) purchased from Aonisite Chemical Trade Co., Ltd. (Tianjin, China). Styrene maleic anhydride (SMA) copolymer (Scripset^®^ 520, Hercules, CA, USA) was applied as dispersant [4]. Diphenylsilane (DPS) (Tianjin Chem., Tianjin, China) was used as rejuvenator, which was a low viscosity and colorless liquid. The bitumen used in this study obtained from Qilu Petrochemical (Linzi, China). The aged bitumen samples with penetration grade of 70.5, 55.4, 47.6, 36.8 and 29.7 were artificially produced by a thin film oven test [10].

### 2.2. Microcapsules Fabrication Processes

The method of fabrication microcapsules containing rejuvenator by coacervation process has been reported in previous works [6,7,8,9,10]. In details, it can be divvied into three steps: (a) SMA was added to 100 mL water at 50 °C and allowed mix for 2 h. Then a solution of NaOH (10%) was added dropwise adjusting its pH value to 10. The above surfactant solution and rejuvenator were emulsified mechanically under a vigorous stirring rate for 10 min using a high-speed disperse machine; (b) The encapsulation was carried out in a 500 mL three-neck round-bottomed flask equipped with a condensator and a tetrafluoroethylene mechanical stirrer. The above emulsion was transferred in the bottle, which was dipped in a steady temperature flume (room temperature). MMF prepolymer was added dropwise with a stirring speed of 500 r·min^−1^. The temperature was increased to 80 °C with a rate of 2 °C·min^−1^; (c) After polymerization for 1 h, the temperature was decreased slowly to ambient temperature. At last, the resultant microcapsules were filtered and washed with pure water and dried in a vacuum oven.

### 2.3. Preparation of Microcapsules/Bitumen Samples

The 50 pen grade aged bitumen was blended with different microcapsules using a propeller mixer for 30 min at 160 °C with a constant speed of 200 r·min^−1^. Hot microcapsules/bitumen mixture was poured on a glass sheet to form thin a film with a thickness less than 200 μm. The samples were keeping under 0 °C waiting for tests.

### 2.4. Microcrack Generation

**Figure 2:**
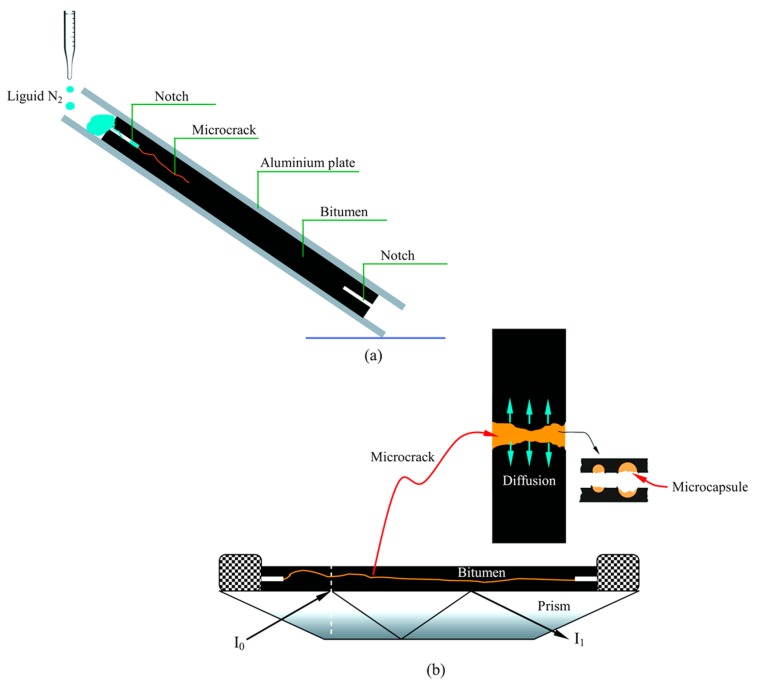
The illustration of FTIR-ATR method testing the diffusion behavior of rejuvenator in bitumen, (**a**) the aluminum plate containing bitumen with notch ends, microcrack generated by liquid N_2_; (**b**) the FTIR-ATR testing prism.

Figure 2a illustrates the microcrack generation process through a self-designed installation. Hot bitumen/microcapsules mixture (100 °C) was poured into the middle of two aluminium plates forming a thin bitumen layer with a thickness of 2 mm. Rectangular notches (length 5 mm) were carved at each end of the bitumen layer. The prepared thin bitumen/microcapsules sample carefully poured with drops of liquid nitrogen (*N*_2_) at one end [8]. Microcrack was quickly generated in this sample because of the low temperature brittleness.

### 2.5. Morphologies Observation

The dried microcapsules were observed by using an Environmental Scan Electron Microscopy (ESEM, Philips XL30, Philips, Amsterdam, The Netherlands) at an accelerated voltage of 20 kV. Self-healing behaviors of bitumen were observed by a fluorescence microscope (CKX41-F32FL, OLYMPUS, Tokyo, Japan). As bitumen is a temperature-sensitive material, the observation is in an environment of 0 °C temperature.

About 2 g MMF-shell microcapsules was mixed in 5 g epoxy resin. After the composite was dried at room temperature, it was carefully cut to obtain the cross-section. The thickness of shells can be measured from the SEM images of cross-section of microcapsules [4]. At least 20 shells of each microcapsule sample were measured and the average data were calculated.

### 2.6. FTIR-ATR Tests

A modified FTIR-ATR (Vector 22, Bruker, Billerica, MA, USA) method [12] was used to continuously monitor rejuvenator diffusion into bitumen. ATR exploits the total internal reflectance of infrared light in a non-absorbing prism. Any absorbing substances in contact with the prism surface will attenuate the internally reflected light and, as a consequence, an infrared absorbance spectrum is obtained, corresponding to a spectrum recorded as if the light passed through the surface layer of the material studied. To determine diffusion rates of a rejuvenator penetrating a bitumen sample, a thin bitumen layer with a microcrack was applied on top of a zinc selenide (ZnSe) ATR prism as shown in Figure 2b. The application was accomplished by gluing brass frames on top of the prism. The bottom layer (the bitumen) was allowed to settle in the heated sample holder at 100 °C for 30 min to obtain absolute contact between the sample and prism, and to avoid initial problems with air bubbles and other types of distress at the beginning of the diffusion test. All thin bitumen layers had a thickness of 2 mm. The temperature was set and infrared absorbance recorded.

## 3. FTIR-ATR Method and Theoretical Framework

**Figure 3:**
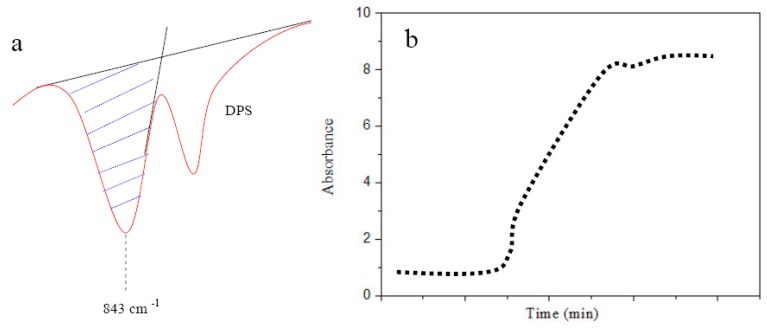
Calculating method of diffusion coefficient, (**a**) the fairly specific absorption band for DPS at 843 com^−1^ selected as a marker band to calculate the diffusion coefficient, the integrated peak area determined as absorbance; (**b**) a logarithmic scale curve of time-absorbance, the sloping line in the horizontal direction determined by the diffusion coefficient.

Normally, FTIR-ATR is used to exploit the attenuation of light reflected internally in a non-absorbing prism, due to energy absorption of an analyte in contact with the reflecting surface. To further enhance the attenuation, and consequently, the absorption spectra, the prism usually has an oblong and trapezoidal shape to allow multiple internal reflections. By using this method, the diffusion through a thin film is detected by quantifying the change in absorbance at wave numbers characteristic for the diffusing substances, thus obtaining the diffusion coefficient (*D*). In these tests, the diffusing substances are flushed over the film while keeping the concentration constant at the surface of the film. A modified FTIR-ATR method [12,13] was applied to determine diffusion rates of rejuvenator penetrating bitumen. Thin layer of bitumen was applied on top of a zinc selenide (ZnSe) ATR prism. The test was started by switching on the temperature control unit, after which the interferograms immediately began to be recorded. The time before reaching the test temperature was monitored to allow corrections for the initial heating period. An insulating cap was placed on top of the ATR sample holder to obtain a more uniform temperature in the sample. The absorbance at certain wave numbers was calculated using the integrated peak area as Figure 3a shown. In this study, a fairly specific absorption band for DPS at 843 cm^−1^ was selected as a marker band to calculate the diffusion coefficient [12]. The calculated absorbance values figured out using a logarithmic time scale (Figure 3b), where the location of the sloping line in the horizontal direction was determined by the *D*.

Diffusion is defined as transport of matter due to random molecular movements termed Brownian motions, and is temperature dependent. For bituminous materials, diffusion takes place at the micro-level and enables the recycled binder to become homogeneous [1,12]. Compatibility between binders is a requirement for creating a homogeneous binder, and is mainly dependent on the nature and distribution of the intermolecular associations. The concept of the diffusion process can be physically verified using a stage extraction method, in which the inner and outer layers of the recycled binder film were extracted separately. It was reported that how the binder stiffness (higher penetration indicates lower stiffness) of the reclaimed material originally varied (the outer layer was stiffer than the inner layer). *Fick’s* law is generally used for the mathematical description of diffusion, and is a simplification of the more general theory of the influence of chemical potential on diffusion as Equation (1) shown,

(1) $$\frac{\partial c}{\partial t}=D\cdot \frac{\partial^{2}c}{\partial x^{2}}$$

where *c* is concentration, *t* is time, *x* is position and *D* is the diffusion coefficient. From Equation (1), it can be seen that diffusion is dependent on the concentration of the diffusion and the diffusion coefficients. At the same time, the diffusion coefficients are different as linear or nonlinear with regards to various models. The Arrhenius equation is a formula for the temperature dependence of reaction rates. The relationship between *D* and temperature can be derived using the heat energy activation approach resulting in Equations (2) and (3),

(2) D=k1⋅ek2T

(3) $$D(T)=D_{0}e^{(d_{1}/T)+d_{2}}$$

where *k*_1_ and *k*_2_ are constants, *d*_1_ and *d*_2_ are constants, and *T* is absolute temperature (unit K). It has been proved that Equation (2) is useful to characterize the diffusion rate in bitumen under the same temperature and Equation (3) can be applied to evaluate the *D* values under different temperatures [12]. Based on the logarithmic scale curve of time-absorbance, curve fitting was performed graphically under different temperature with various microstructure of microcapsules/bitumen (mean size, thickness, content). A number of measures were undertaken to increase the reliability of modeling.

## 4. Discussion and Results

### 4.1. Microcapsules/Bitumen Samples 

**Figure 4:**
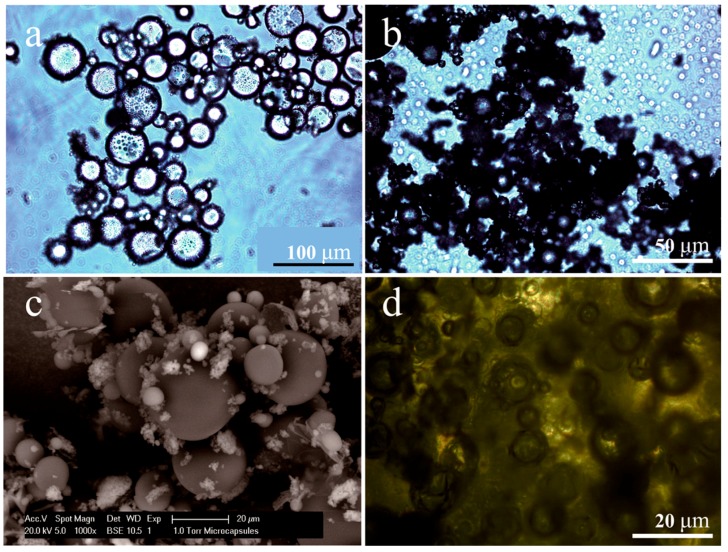
Morphologies of microcapsules and microcapsules/bitumen composite, (**a**,**b**) optical morphologies of microcapsules; (**c**) ESEM morphologies of microcapsules; (**d**) fluorescence microscope morphology of microcapsules/bitumen composite at 25 °C.

Usually, microcapsule product has three main parameters: morphology, mean size and shell thickness. Figure 4a,b shows the optical morphologies of microcapsules in emulsion. It can be seen that the MMF prepolymer have polymerized on the exterior of the oil droplets forming shells. This polymerization is due to the help of hydrolyzed SMA molecules, which are adsorbed at the interfaces of oil droplets; these molecules readily undergo directional arrangement such that the hydrophobic groups are positioned in the oil droplets while the hydrophilic groups extend out of the droplets [4]. Figure 4c shows ESEM morphologies of dried microcapsules with a mean size about 20 μm. They have a regular globe shape and smooth surfaces. The mean size values can be controlled by the method of regulating the emulsion speed during polymerization. Figure 4d shows a typical fluorescence microscope morphology of microcapsules /bitumen composite at 25 °C. The microcapsules retain their compact shell structure during mixing with hot bitumen, maintaining their original shape and smooth surface. It is evident that the thermal effect did not break the shells. This phenomenon is consisted to previously reported result that microcapsules could resist a temperature of about 180 °C without being broken.

The shell thickness values were measured by observing the cross-section ESEM morphology of microcapsules. Normally, the shell thickness is dominated by the amount of shell material during the coacervation process, a lower core/shell ratio (weight ratio of core/shell) leads to a higher shell thickness value [6]. To simplify the complexity, the microcapsules have the same core/shell ratio of 2:1. Table 1 lists six microcapsule samples, coded as M-1, M-2, M-3, M-4, M-5 and M-6, with various shell thickness and mean size values. It must be mentioned that shell thickness and mean size of microcapsules both have a normal distribution according to the in situ polymerization. Each data was an average value of five testing results. The samples have the shell thickness in a range of about 1.2 μm–2.7 μm. The thickness of asphalt binder between aggregates is less than 50 μm, the size of microcapsules containing rejuvenators must be smaller than 50 μm to avoid being squeezed or pulverized during asphalt forming [4]. Therefore, the samples have the mean size values about 10 and 20 μm.

### 4.2. Observation of the Microcrack Generation and Diffusion Behaviors

**Figure 5:**
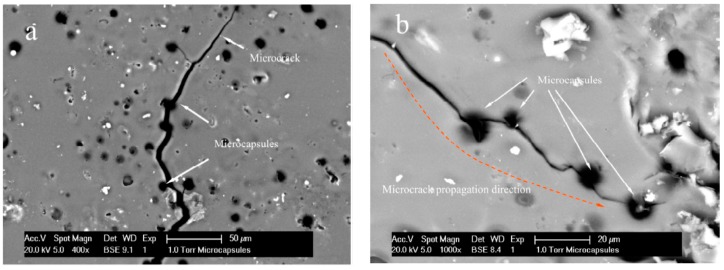
ESEM morphologies of microcracks in bitumen, (**a**) a microcrack propagation direction; (**b**) a microcrack punctured several microcapsules.

Before investigating internal diffusion behaviors, it must be confirmed that the healing of microcapsule/bitumen composites is coming from the microcapsules. In other words, the microcapsules can be broken and the encapsulated rejuvenator can be released and diffuse into bitumen. For this purpose, a microcrack therefore was generated by liquid N_2_ with a width less than 10 µm to stimulate a self-healing process. Figure 5a,b shows the microcrack propagated successfully and pierced microcapsules along the development track and the shells of microcapsules were broken by a microcrack. Especially in Figure 5b, the diffusion behavior can be observed around the broken microcapsules. In previous work [9,10], it has been reported that the liquid rejuvenator can leak out from microcapsules and flow into the microcrack, and then rejuvenator spreads along the whole microcrack through capillarity. It can also be demonstrated in Figure 5 that a microcrack can pierce several microcapsules along the propagation trace. Thus, enough rejuvenator can be supplied diffusing into the aged bitumen.

**Figure 6:**
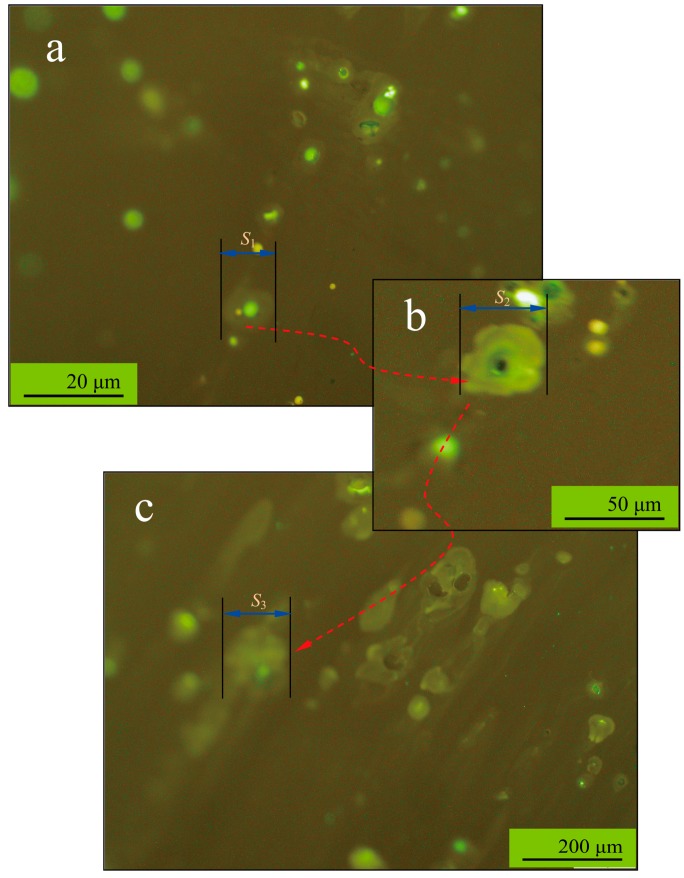
Fluorescence microscope morphologies of microencapsulated rejuvenator diffusing into bitumen under 25 °C at different time, the diffusion diameters of rejuvenator from one microcapsule: (**a**) *S*_1_ at 30 min; (**b**) *S*_2_ at 60 min, and (**c**) *S*_3_ at 90 min.

Figure 6a–c shows the fluorescence microscope morphologies of microencapsulated rejuvenator diffusing into bitumen under 25 °C at a different time. When the microcapsule was broken, the rejuvenator could quickly disperse around the shell. Mass transfer by molecular diffusion is one of the basic mechanisms in many branches of science. Molecular diffusion is a transport property which controls the rate of mass transfer of species in a medium. However, the diffusion behaviour of bitumen has a significant relationship with temperature, time, and viscosity of the diffusion medium, diffusant size and polarity [15]. To simplify this diffusion process, the diffusion behaviour was identified by measuring the diffusion diameters (*S*_1_, *S*_2_ and, *S*_3_) of rejuvenator from one microcapsule at different time of 30, 60 and 90 min. By comparing the diameter values of diffusion areas of these microcapsules, the diffusion direction can be pointed out and the diffusion rate can be roughly estimated. With the time increasing, the diffusion area gradually becomes larger from 10 μm to 100 μm. Furthermore, the diffusion kinetic behavior, which is determined by the rate of mass transfer between the rejuvenator and aged bitumen under given conditions, can be calculated based on repeated measurement. At the same time, the diffusion coefficient between liquid-solid phases can be deduced based on the factors of liquid molecular structure, solid structure, temperature and pressure.

### 4.3. Microcapsules Contents Dependency of Diffusion

**Figure 7:**
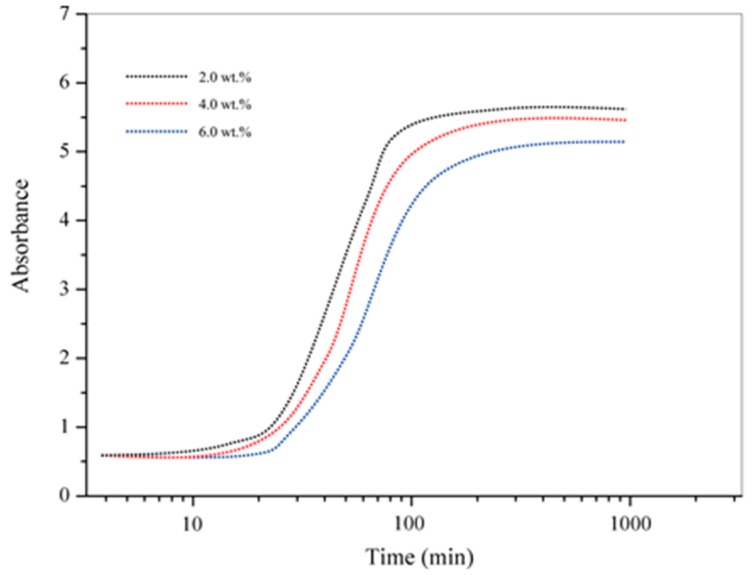
Absorbance-time curves of 843 cm^−1^ band at 30 °C with the microcapsules contents of 2.0, 4.0 and 6.0 wt %.

It must be noted that the diffusion coefficients of the asphalt binder in this study are not the true values because the measurement of diffusion rates of selected substance is only a marker. By carefully comparing the diffusion rates varying parameters, such as microcapsules content (*W_m_*), microcapsules mean size (*δ_size_*) and shell thickness (*δ_thick_*), the results can be applied to analyze the microstructure of asphalt binders. It is necessary to be aware the fact that the diffusion molecules (DPS) will affect the diffusion media and thereby change the diffusion rates [12]. Figure 7 shows the absorbance-time curves of DPS at 843 cm^−1^ band under 30 °C during 1000 min in bitumen samples with the *W_m_* values of 2.0, 4.0 and 6.0 wt %. All the additive microcapsules were the sample M-1. As the microcapsules have the same core/shell ratio of 2:1, DPS weight content in microcapsules/bitumen composite can be calculated as Equation (4),

(4) $$W_{DPS}=\frac{2}{3}\times W_{m}$$

where *W_DPS_* is the DPS weight content, *W_m_* is the microcapsule weight content. The logarithmic time scale reveals that the diffusion curves fit the theoretical curves well, which is consisted with the reported results [13]. In this way, it is possible to indicate by comparing the slopes of curves, as expected, that higher concentration of DPS increases the diffusion rates. It means that the *W_m_* had affected the diffusion coefficient. It has been well known that one important capability of rejuvenator is to soften the age bituminous binders and thereby accelerate the diffusion process [8].

**Figure 8:**
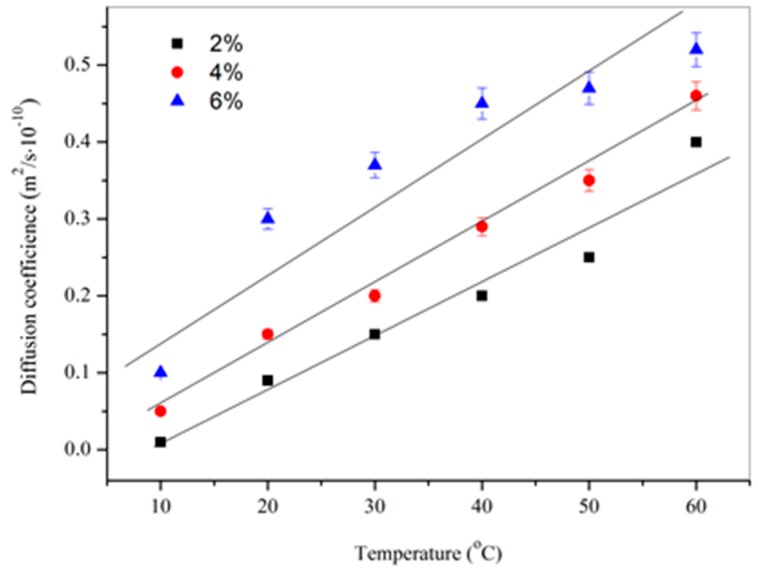
Diffusion coefficient values of microcapsules (sample of M-1) in bitumen with contents of 2%, 4% and 6% under a temperature range of 10 °C–60 °C.

Another factor can enhance the diffusion rate is the temperature, which will promote the molecules movement speed [16]. Figure 8 shows the diffusion coefficient values of DPS in bitumen samples mixing with microcapsules (sample of M-1) with contents of 2%, 4% and 6% under a temperature range of 10 °C–60 °C. With the temperature increasing, the diffusion coefficient values also liner increased sharply. Moreover, more microcapsules mixed in bitumen had enhanced the diffusion coefficient values in accord with the results in Figure 7.

### 4.4. Mean Size and Shell Thickness Dependency of Diffusion

The self-healing process of aged bitumen based on the microcapsules containing rejuvenator is a series of movement of rejuvenator, including the releasing from broken microcapsules, the capillary movement in microcracks and the diffusion movement in bitumen [8]. This complex mechanism has been reported [9] in previous works by analyzing the microstructure of microcapsules/bitumen samples. Therefore, the microstructure, such as mean size (*δ_size_*) and shell thickness (*δ_thick_*) of microcapsules will greatly influence the shell strength [5]. Shell strength determines the damage rate of microcapsules by the microcracks [9]. Higher shell strength will resist the tip stress of a microcrack keeping the integrality a microcapsule. The result means less rejuvenator can be released and then soften the bitumen.

**Figure 9:**
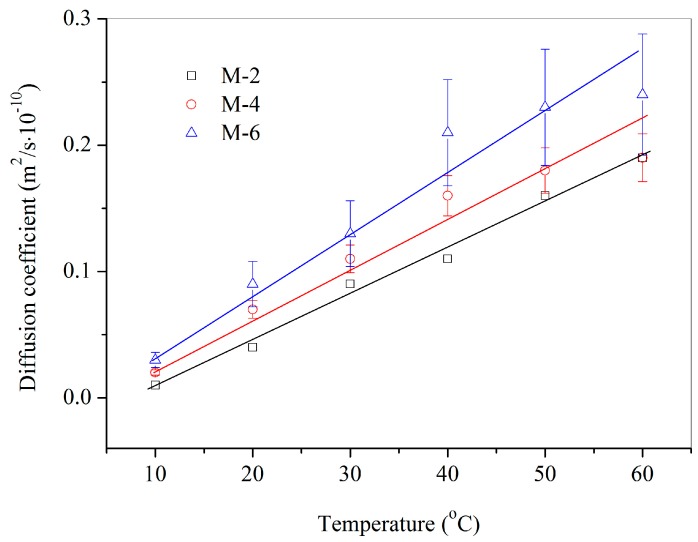
Diffusion coefficient values of microcapsules (content of 6%) in bitumen with different mean size values (samples of M-2, M-4 and M-6) under a temperature range of 10 °C–60 °C.

**Figure 10:**
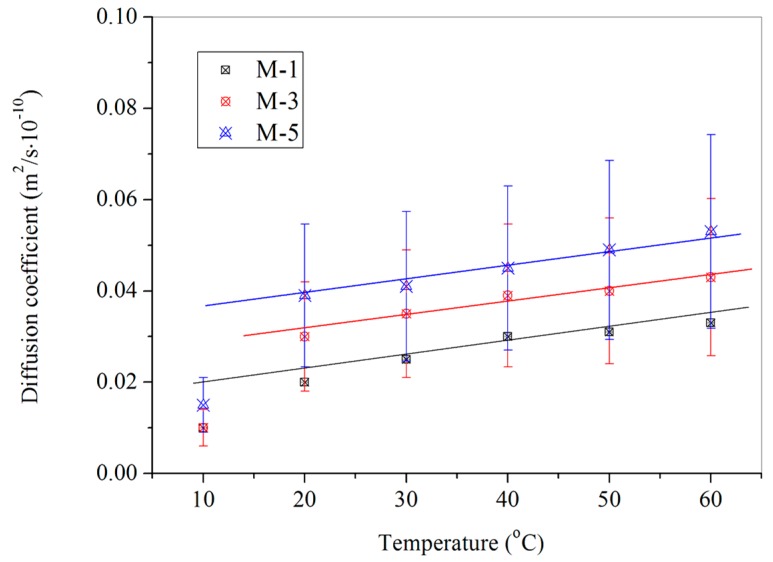
Diffusion coefficient values of microcapsules (content of 2%) in bitumen with different shell thickness values (samples of M-1, M-3 and M-5) under a temperature range of 10 °C–60 °C.

Figure 9 shows the diffusion coefficient values of DPS in bitumen samples with 6% microcapsules under a temperature range of 10 °C–60 °C. These microcapsule samples (M-2, M-4 and M-6) had different mean sizes. It can be seen that the diffusion coefficient values have a linear growth with the increasing of temperature. It is consistent with the above conclusions as shown in Figure 8. On another hand, it is found that larger microcapsules lead to a higher diffusion rate under the same temperature for the various samples. Under temperature of 10 °C, bitumen samples mixing with M-2, M-4 and M-6 have a nearly same diffusion coefficient value. However, the diffusion coefficient value of M-6/bitumen sample is nearly 130% of M-2/bitumen. In previous work, it has been proved that larger microcapsules can enhance the encapsulation ratio of core materials. The above conclusion may be attributed to the reason that larger microcapsules own more rejuvenator. Figure 10 shows the diffusion coefficient values of bitumen samples mixing with 2% microcapsules of M-1, M-3 and M-5 under a temperature range of 10 °C–60 °C. M-1, M-3 and M-5 have the different shell thickness values. With temperature increasing, the diffusion coefficient also has a linear growth. However, fitting straight lines for various microcapsules/bitumen samples are close to coincidence. It indicates that the shell thickness values do not greatly influence the diffusion coefficient. This conclusion can be drawn that the microcapsules samples with different shell thickness values have a nearly same amount of rejuvenator. It also can be deduced that the three microcapsules in bitumen may have the same damage ratio by microcracks. The microcapsules were fabricated in this study by an in situ polymerization, which lead the shell thickness had a size with a normal distribution. The shell of microcapsules is a tiny polymeric membrane. Moreover, all microcapsules have the same core/shell ratio in this study. The normal distribution may weaken the influence of shell thickness on the average data of shell thickness [17]. In other words, this normal distribution also weakens the discrepancy of damage ratio for different microcapsules in bitumen by microcracks.

### 4.5. Aged Degree Dependency of Diffusion

**Figure 11:**
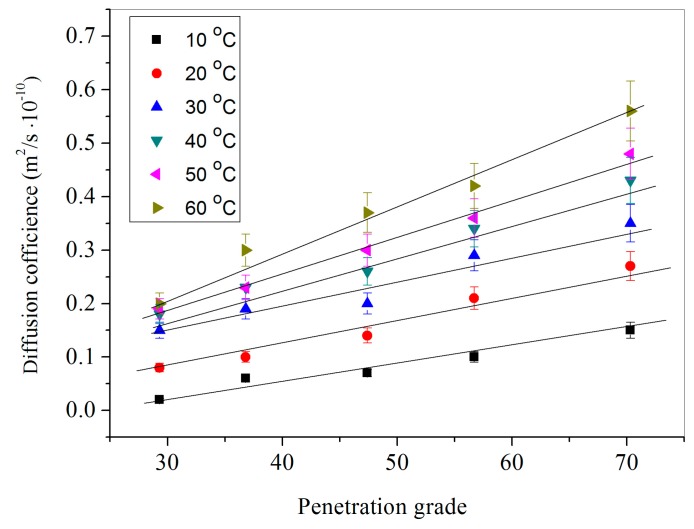
Diffusion coefficient values of microcapsules (content of 6%) in bitumen with different penetration grade values (29.7, 36.8, 47.6, 55.4 and 70.5) under a temperature range of 10 °C–60 °C.

Figure 11 shows the diffusion coefficient values of DPS in bitumen with 6% microcapsules (M-1) under a temperature range of 10 °C–60 °C. Various bitumen samples were applied with different penetration grade values of 29.7, 36.8, 47.6, 55.4 and 70.5. It can be seen that the temperature enhances the diffusion rate for the same bitumen microcapsules/bitumen sample. Under the same temperature, the diffusion coefficient values have a linear growth. The diffusion coefficient values do not have a significant growth under temperature of 10 °C. However, the fitting linear at 60 °C has the largest slope, which indicates that DPS has a highest diffusion rate. This conclusion is consistent with the reported results that the rejuvenator has a higher diffusion rate in higher penetration grade of bitumen [12]. The reason is that small molecules of rejuvenator can easily move through the bitumen with a relatively loose structure [18]. Bitumen is one kind of colloid material and the large molecules together by intermolecular force and form a network structure. The network structure might be the principal barrier for the diffusion of rejuvenator molecules. At the same time, the addition of rejuvenator can break the network structure and accelerate the molecular diffusion. The content of large molecules of age bitumen was large, therefore, rejuvenator had a higher diffusion rate in bitumen with a relatively larger penetration grade.

### 4.6. Preliminary Model of Diffusion Coefficient

All the above results indicate that the microstructure of microcapsules greatly influent the diffusion behaviors based on the concentration of released rejuvenator. Therefore, it is meaningful research to supply a semi-experimental model of diffusion coefficient. In previous works, the self-healing efficiency has been evaluated by mechanical tests such as modified beam on elastic foundation method [9] and a repetitive direct tension method [19]. Direct methods involve the compositional analysis of mechanical properties of microcapsules/bitumen before and after a designed self-healing process. Basically, it was deduced that the mechanism of self-healing based on microcapsules technology was a rejuvenator diffusion process [9,10]. In other words, a model of diffusion coefficient can be applied as a guide to indirectly predict the self-healing efficiency. It must be noted that the diffusion coefficient concept in this study is different to the normal rejuvenator diffusion coefficient, because the microcapsules have great effects on the diffusion process. The model is only a semi-experimental model, which can expose some microstructure information about asphalt. 

In the microcapsules/bitumen material system, diffusion coefficient (*D*) can be considered as a function as Equation (5),

(5) $$D=f(\delta_{size},\delta_{thick},R_{c/s},D_{r},W_{m},A_{d},T)$$

where *R_c/s_* is the core/shell material ratio, *D_r_* is the damage rate of microcapsules in bitumen, and *A_d_* is the age degree of bitumen. The *A_d_* is defined as the percentage of penetration degree between aged bitumen at time (*t*) and the original bitumen. *R_c/s_* value is a constant in this study. The influence of *δ_thick_* is ignored in this model because it does not play a decisive role for diffusion behaviors as the results in Figure 10. In contrast, the *D_r_* is a main factor affecting the amount of rejuvenator diffusing into aged bitumen. It has been proved in Figure 7 that the diffusion curves fit the theoretical curves well with a logarithmic time scale. Based on the above consideration, the Equation (2) can be written as Equation (6),

(6) $$\text{ln}\;D(T)=\lambda_{1}+\lambda_{2}/T$$

where *λ*_1_ and *λ*_2_ are constants. Introducing the relation of the above parameters into this formula,

(7) $$\text{ln}\;D=\lambda_{1}{A_{d}}^{\varphi_{1}}+\lambda_{2}{\left(\delta_{size}\delta_{thick}R_{c/s}\right)}^{\varphi_{2}}{\left(D_{r}W_{m}\right)}^{\varphi_{3}}{A_{d}}^{-\varphi_{4}}/T$$

where *ϕ*_1_, *ϕ*_2_, *ϕ*_3_ and *ϕ*_4_ are constants. *ϕ*_2_ is determined by the microstructure of microcapsules, *ϕ*_3_ is determined by the released amount of oily rejuvenator in diffusion process, and *ϕ*_1_ and *ϕ*_4_ is determined by the aged degree of bitumen. The above three parameters will be given in future work by mathematical fitting with a large number of experimental data. Based on the above experimental results, this model will be a guide to the construction and application of self-healing bitumen using microcapsules.

## 5. Conclusions

In this study, diffusion behaviors of microencapsulated rejuvenator in aged bitumen were evaluated by a FTIR-ATR method. The core material of microcapsules used as rejuvenator was diphenylsilane (DPS), its fairly specific absorption band was selected as a marker band to calculate the diffusion coefficient. From the mentioned preliminary results, the following conclusions can be concluded.

1. The microstructure affected the D values of DPS in aged bitumen. A higher mean shell thickness decreased the D values because of the decrement of damage probability of microcapsules under the same content.
2. With the same microcapsule sample in bitumen, the D values presented a trend of linear increase when the content of microcapsules was increased. All these results indicated that the microstructure greatly influent the diffusion behaviors based on the concentration of released rejuvenator.
3. A preliminary model of diffusion behaviors of microencapsulated rejuvenator in bitumen was given based on the Arrhenius equation. This model considered the influence factors of microstructure, the amount of released rejuvenator and the age degree of bitumen. It is a guide to the construction and application of self-healing bitumen using microcapsules.

## Figures and Tables

**Table 1:** Microcapsule samples with various shell thickness and mean size.

Microcapsule Samples	Shell Thickness (μm)	Mean Size (μm)
M-1	1.2 ± 0.6	10 ± 0.45
M-2	2.6 ± 0.5	10 ± 0.40
M-3	1.5 ± 0.6	20 ± 1.53
M-4	2.7 ± 0.7	20 ± 1.53
M-5	1.6 ± 0.7	30 ± 3.52
M-6	2.7 ± 0.6	30 ± 3.08

## Nomenclature

Diffusion coefficient	*D* (m^2^/s)
Concentration	*C* (L/m^3^)
Temperature	*C* (L/m^3^)
Coordiante direction	*x* (m)
Time	*t* (s)
Diphenylsilane	DPS
Microcapsule weight content	*W_m_* (wt %)
DPS weight content	*W_DPS_* (wt %)
Microcapsules mean size	*δ_size_* (m)
Microcapsules shell thickness	*δ_thick_* (m)
DPS weight content	*W_DPS_* (wt %)
Core/shell material ratio	*R*_c/s_
Damage rate of microcapsules in bitumen	*D_r_* (%)
